# A Systems Modeling Approach to Forecast Corn Economic Optimum Nitrogen Rate

**DOI:** 10.3389/fpls.2018.00436

**Published:** 2018-04-13

**Authors:** Laila A. Puntel, John E. Sawyer, Daniel W. Barker, Peter J. Thorburn, Michael J. Castellano, Kenneth J. Moore, Andrew VanLoocke, Emily A. Heaton, Sotirios V. Archontoulis

**Affiliations:** ^1^Department of Agronomy, Iowa State University, Ames, IA, United States; ^2^CSIRO Agriculture, St Lucia, QLD, Australia

**Keywords:** corn, economic optimum N rate, forecast, modeling, APSIM, in-season nitrogen management, nutrient recommendation

## Abstract

Historically crop models have been used to evaluate crop yield responses to nitrogen (N) rates after harvest when it is too late for the farmers to make in-season adjustments. We hypothesize that the use of a crop model as an in-season forecast tool will improve current N decision-making. To explore this, we used the Agricultural Production Systems sIMulator (APSIM) calibrated with long-term experimental data for central Iowa, USA (16-years in continuous corn and 15-years in soybean-corn rotation) combined with actual weather data up to a specific crop stage and historical weather data thereafter. The objectives were to: (1) evaluate the accuracy and uncertainty of corn yield and economic optimum N rate (EONR) predictions at four forecast times (planting time, 6th and 12th leaf, and silking phenological stages); (2) determine whether the use of analogous historical weather years based on precipitation and temperature patterns as opposed to using a 35-year dataset could improve the accuracy of the forecast; and (3) quantify the value added by the crop model in predicting annual EONR and yields using the site-mean EONR and the yield at the EONR to benchmark predicted values. Results indicated that the mean corn yield predictions at planting time (*R*^2^ = 0.77) using 35-years of historical weather was close to the observed and predicted yield at maturity (*R*^2^ = 0.81). Across all forecasting times, the EONR predictions were more accurate in corn-corn than soybean-corn rotation (relative root mean square error, RRMSE, of 25 vs. 45%, respectively). At planting time, the APSIM model predicted the direction of optimum N rates (above, below or at average site-mean EONR) in 62% of the cases examined (*n* = 31) with an average error range of ±38 kg N ha^−1^ (22% of the average N rate). Across all forecast times, prediction error of EONR was about three times higher than yield predictions. The use of the 35-year weather record was better than using selected historical weather years to forecast (RRMSE was on average 3% lower). Overall, the proposed approach of using the crop model as a forecasting tool could improve year-to-year predictability of corn yields and optimum N rates. Further improvements in modeling and set-up protocols are needed toward more accurate forecast, especially for extreme weather years with the most significant economic and environmental cost.

## Introduction

**Figure 1:**
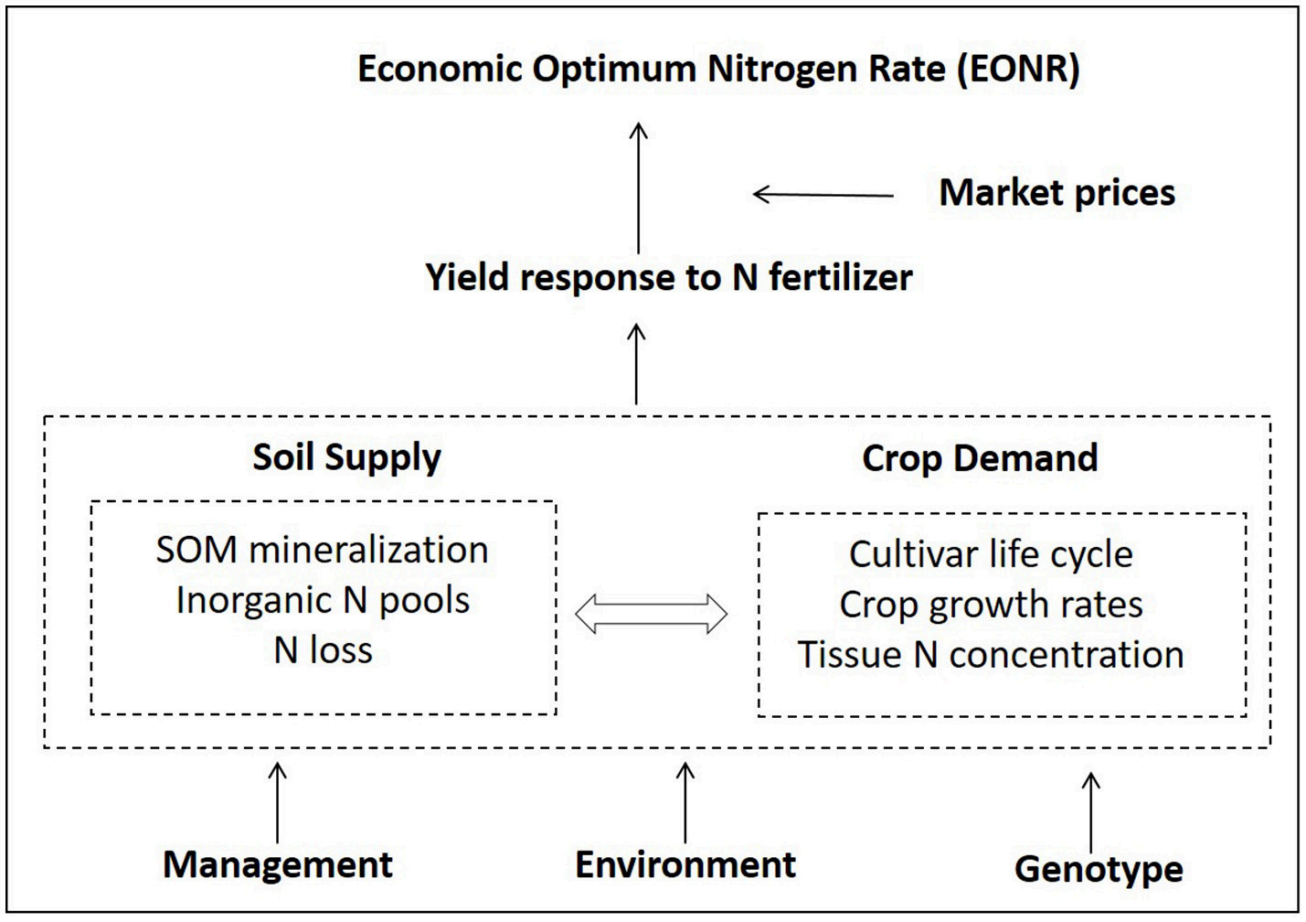
Overview of the main factors influencing the economic optimum nitrogen fertilizer (EONR) rate and their interactions. Soil organic matter (SOM).

Over and under fertilization of nitrogen (N) in corn production affects the farmer's profitability and the environment (Shanahan et al., 2008). Predicting the economic optimum N rate (EONR) before crop planting is an ongoing research effort. The challenge persists because of multiple dynamic factors influencing the EONR. In brief, genotypic inputs (cultivars), environment (soil × weather, especially rainfall and its distribution), and management choices (tillage, N application time, etc.) affect soil and crop processes in various ways. The result of all these dynamic processes and their interactions (soil supply vs. crop demand) determine the yield at any N fertilization level (Figure 1). There are several examples in the literature where a single component of the system was studied in detail without acknowledging other system's components and their inherent feedbacks (effect of tillage, effect of residue removal, Kwaw-Mensah and Al-Kaisi, 2006; Coulter and Nafziger, 2008). Several tools and methodologies have been developed over time to assist farmers with N rate decisions (e.g., yield goal approach, Stanford, 1973; soil nitrate test, Bundy and Andraski, 1995; Shapiro et al., 2008), while other new tools such as sensor technologies and simulation models are currently being developed and tested (Scharf, 2015; Banger et al., 2017).

Most of today's N-rate decision tools focus on a single component of the soil-plant system to predict EONR rather than utilizing all of the N dynamics and interactions that occur among processes in the system (Figure 1; Arbuckle and Lasley, 2013). For example, the single time soil testing approach around corn at 6th leaf stage provides an estimate of soil N supply (Magdoff et al., 1984; Blackmer et al., 1989; Binford et al., 1992). However, the prediction accuracy and usage of this approach is low because the soil nitrate varies greatly in time and space, especially in rainfed production regions with unpredictable rain events (Jemison and Lytle, 1996; Ma et al., 2007; Arbuckle and Lasley, 2013). Other examples are crop sensors and remote sensing technologies that provide an estimate of crop N status during the season (Yuan et al., 2016). Although promising as a N diagnostic tool this approach has not yet been widely adopted by farmers (Mamo et al., 2003; Scharf et al., 2005; Hawkins et al., 2007) because they typically delay N application until the plant N status can be reliably determined. That requirement increases the risk of not being able to apply N or adds extra application costs (Van Es et al., 2006; Tremblay et al., 2012; Franzen et al., 2016).

Another N tool, the yield goal (Stanford, 1973; Stanford and Legg, 1984), requires inputs such as grain yield, N concentration, nitrogen use efficiency, and N credits coming from manure, legume crops, and soil organic matter to determine corn N rates. These inputs are difficult to estimate because they are derived properties (outputs) of many interactive processes occurring simultaneously within the soil-plant-atmosphere system (Figure 1). Thus, farmers use prior knowledge or guesswork to provide these inputs. According to Lory and Scharf (2003) and Shanahan (2011), the yield goal approach usually results in over fertilization as a form of “insurance” against uncertain soil N supply. In contrast, the Maximum Return To Nitrogen approach (MRTN; Sawyer et al., 2006) requires simple inputs such as location, rotation, crop and fertilizer prices. By using an extensive experimental network of derived N-response datasets across the USA Midwest, it provides farmers with a N-rate recommendation per geographical areas (Sawyer et al., 2006).

In contrast to the aforementioned tools, process-based cropping system models that account for different soil-crop processes, and their interactions with management, cultivar and environmental conditions (Figure 1), have demonstrated capabilities explaining causes of EONR variability and perform scenario analyses (Thorp et al., 2007; Gowda et al., 2008; Nangia et al., 2008; Puntel et al., 2016). However, most of the model applications in N research have been applied ex-post (after harvest), which is of limited use to farmers (Kersebaum et al., 2005; Nendel et al., 2013).

Successful crop yield forecasting approaches using process-based models and historical weather in the USA (Morell et al., 2016; Togliatti et al., 2017) and Australia (Carberry et al., 2009), have indicated the potential to complement the explanatory power of cropping system models with the forecasting component that is needed for N decisions. Theoretically, by running a process-based model for different N rates using actual and historical weather, data the model can provide end-of-season yields (the yield-N response curve) at any time during the growing season. Thus, the EONR could be estimated as early as planting time, providing a new approach to make N rate decisions and supporting information on crop yields, N supply, and N demand (Figure 1). To our knowledge, the validity of this approach and the uncertainty around the year-by-year (annual) EONR prediction at planting and during the growing season have not been previously investigated.

A critical aspect in crop yield forecasting (and ultimately N-forecasting) is the use of weather information to drive model simulations. Traditionally, historical weather data is used to fill the unknown weather for the remainder of the growing season and calculate yield probabilities (Hammer et al., 1996; Quiring and Legates, 2008). Thus, forecasting yield and optimal N-rate at planting time is a challenge given the uncertainty in weather. As weather information becomes available during the season, the uncertainty around crop yield prediction decreases, but it follows different patterns from year to year and cropping systems (Archontoulis et al., 2016; Togliatti et al., 2017). Furthermore, use of analogous historical weather years (i.e., weather with similar precipitation or temperature patterns as the year to be forecasted) instead of using 35-year record has been used as an approach to improve yield forecast (i.e., Hammer et al., 2001; Hansen et al., 2004). The time of the forecast may also affect yield and EONR predictions and uncertainty patterns.

In this study, we tested the hypothesis that the use of a calibrated cropping system model coupled with an assembly of actual and historical weather datasets can predict EONR as early as planting time with similar accuracy as the prediction at harvest with known weather (ex-post). We build upon Puntel et al. (2016) in which the APSIM model was calibrated using 16 years of corn yield response to N data from two crop rotation systems at a site in central Iowa, USA. Our objectives were to: (1) evaluate the accuracy and uncertainty of corn yield and EONR predictions at four forecast times (planting time, 6th and 12th leaf, and silking crop physiological stages) compared with the observed and simulated values at harvest; (2) investigate whether the use of selective historical weather records (e.g., years with similar precipitation) will increase accuracy of yield and EONR predictions as opposed to the 35-year historical record; and (3) quantify value added by the crop model in predicting annual EONR and yields using the site-mean EONR and the yield at the EONR (average of yearly values) to benchmark predicted values.

## Methods

### Experimental data and site description

We used 16 years (1999–2014) of corn yield response to N fertilizer rate data from a field experiment conducted in central Iowa, USA (details in Puntel et al., 2016). The experiment was designed to study the effect of five N fertilizer rates (0, 67, 134, 201, and 268 kg N ha^−1^) on corn yield in continuous corn (CC) and corn following soybean (SC) cropping systems. Application of N was either pre-plant or side-dress; see details in Puntel et al. (2016). Corn grain yield was reported at 15.5% moisture content. The corresponding EONR values per year and rotation were calculated using measured yields (see section Estimation of the Annual Economic Optimum Nitrogen Rate). The weather at the experimental site is humid continental with warm rainy summers with an average annual precipitation of 900 mm and annual temperature of 9°C. Over the 16-year experimental period, crops experienced warm and wet conditions (3 years), cool and wet conditions (3 years), warm and dry conditions (5 years), and cool and dry conditions (5 years; Figure S1a). The soil at the site is a deep fertile loamy (Clarion soil series) with topsoil organic matter of 3.4% and profile plant available water of 250 mm.

### The APSIM modeling platform, set up, and calibration

The APSIM model is an open-source advanced simulator of agricultural systems that combines several process-based models in a modular design (Holzworth et al., 2014). In this study, we used the recently calibrated version of the model for this site with no additional changes (see Puntel et al., 2016 for detailed calibration information). Model performance is also provided in this study (see yield predictions at maturity, results section). The simulation process was continuous, starting in 1999 and ending in 2014 without annual re-initialization of inputs to capture carryover effects on soil N, water, residue, and soil organic matter dynamics from 1 year to the next. The following APSIM models were used: corn and soybean crop models (Keating et al., 2003), Soil N (soil N and C cycling model; Probert et al., 1998), SWIM (soil water model using the Richard equation and fluctuated water tables; Huth et al., 2012); SURFACEOM (residue model; Probert et al., 1998; Thorburn et al., 2001; Thorp et al., 2005), soil temperature (Campbell, 1985), and the following management rules: planting, harvesting, fertilizer, tillage, and rotations (Keating et al., 2003).

### Forecasting yields and EONR

To forecast yields and EONR in each study year (1999–2014) we combined actual weather data up to a specific crop stage and historical weather data thereafter (Figure S2). The following forecast times were considered in this study: planting, 6th leaf (V6), 12th leaf (V12), and silking (R1; Abendroth et al., 2011). We selected these times because farmers in rainfed production regions can make delayed N applications during vegetative and early reproductive stages (R1) without a significant negative effect on yield (Scharf et al., 2002). In this environment, there is not much evidence of yield response to N fertilizer application after corn silking, thus we did not explore yield and N forecasts during grain filling period.

Except for planting time, which only includes observed weather data until the planting date, the rest of the forecast times used observed in-season weather data until the forecast time (Figure S2). Historical daily weather data from 1980 to 2015 (35 years, maximum available for this site) was obtained from the Iowa Environmental Mesonet (2014) to fill the unknown weather. The combination of 35 years of simulations, four forecast times, 16 study years, five N rates, and two crop rotations resulted in more than 22,000 simulations. We calculated the yearly mean and standard deviation of yield and EONR from the 35 simulated values per year and forecasting time similar to Togliatti et al. (2017). The simulated site-mean EONR was calculated by averaging individual annual estimates of EONR and yield at EONR (YEONR) for each rotation, and then calculating the associated standard deviation (*SD*; across years mean).

Once the simulations were completed, we grouped the data to represent the following five weather scenarios and quantify the impact on yield and EONR forecasts:

- Use simulated yields from calendar years with similar *annual* precipitation and temperature patterns as the year of interest (e.g., 2010 and 1984). These groups of years can be found in Figure S1a.
- Use simulated yields from calendar years with similar *summer (June to August)* precipitation and temperature patterns as the year of interest. These groups of years can be found in Figure S1b.
- Use simulated yields from the five calendar years prior to the study year (e.g., for 2010 we used 2005–2009 years).
- Use simulated yields from the 10 calendar years prior to the study year.
- Use simulated yields from the 20 calendar years prior to the study year.

Scenarios I and II were investigated because previous findings showed that the used of analogous weather years instead of the full weather record could increase accuracy of yield predictions and thus, derived EONR predictions (Hammer et al., 2001; Hansen et al., 2004). Scenarios III to V were investigated to quantify the impact of using limited amount of weather data because long term (35-years) weather data are not available in every location (Hansen et al., 2004; Grassini et al., 2015).

### Data analysis

#### Estimation of the annual economic optimum nitrogen rate

The relationship between observed or simulated yield and the five N rates was fit using the quadratic and quadratic-plus-plateau model following the methodology described in Puntel et al. (2016). Models were deemed significant at *p* < 0.05 and the equations with the smallest sums of squares and largest *R*^2^ were selected. The EONR and YEONR was calculated from the N response equations by setting the first derivative of the fitted response curve equal to a common price ratio of 5.6:1 N: corn grain price (US$ kg^−1^ N: US$ kg^−1^ grain) ratio during the study years (Cerrato and Blackmer, 1990; Bullock and Bullock, 1994). Variations in N: grain price ratios during the period of this study (1999–2014) were not taken into consideration because of its potential confounding effect on the simulated EONR and YEORN evaluation. Different price ratios will need to be considered for historical evaluation (Amatya et al., 2008; Sawyer, 2015).

Both the observed EONR and YEONR for each year are presented in Puntel et al. (2016).

#### Statistical evaluation of model performance

To evaluate the APSIM model simulations goodness of fit, we used graphical and statistical methods. For the statistical evaluation, we computed the root mean square error (RMSE), and the relative root mean square error (RRMSE; see equations in Archontoulis and Miguez, 2015) between observed and predicted values. The lower the value of RMSE and RRMSE the better the model performed. In this study, we considered RRMSE ≤15% as “good” agreement; 15–30% as “moderate” agreement; and ≥30% as “poor” agreement (Liu et al., 2013; Yang et al., 2014).

We quantified the accuracy of yield and EONR predictions by comparing the closeness agreement between the simulated and observed means. The accuracy of EONR prediction was considered good when the error was < ± 30 kg N ha^−1^. This threshold represents the historical and current suggested N fertilization rate range for corn (Voss and Shrader, 1979; Sawyer et al., 2006).

The uncertainty around the simulated yields and EONR was calculated as the standard deviation of the 35 estimates (Togliatti et al., 2017). The standard deviation characterized the range of values within which the mean prediction is asserted to lie. We used the standard deviation among forecasting times to evaluate the impact of known weather on the uncertainty around the mean prediction for EONR and yield. Finally, we evaluated the proximity of the observed EONR and yield values to the acceptable range within the standard deviation.

To calculate the site-mean EONR and YEONR, we averaged annually observed values for each rotation similar to Puntel et al. (2016). These site-mean values were used to benchmark model predictions, i.e., above, below or at average. We then counted the number of years in which the model correctly predicted the direction being above, below or at average of EONR and YEONR values at planting time. In each year, we calculated the absolute differences between the observed and the predicted EONR and we counted the years with an average difference of ± 30 kg N ha^−1^.

We also examined APSIM predictions of EONR in extreme N rate years defined as years where the observed EONR is at least 30 kg N ha^−1^ above or below the site-mean EONR. We specified three categories: (1) 30 kg N ha^−1^ greater than the site-mean EONR (high N need), (2) within ± 30 kg N ha^−1^ of the site-mean EONR, and (3) 30 kg N ha^−1^ lower than the site-mean EONR (low N need). In addition, we explored the performance of the model by measuring the agreement (correlation, *R*^2^) between the observed and predicted EONR and YEONR at each forecasting time and by weather conditions based on Figure S2.

#### Statistical evaluation of weather scenarios

The impact of the weather scenarios on yield and EONR predictions was evaluated by calculating the RRMSE for each scenario and then subtracting the RRMSE value of each scenario from the RRMSE of the standard approach using the 35-years of weather data. A positive difference means that a scenario (I–V) performed worse than the 35-year standard approach and a negative difference means that selection of weather years was better than the 35-year standard approach.

## Results

### Yield predictions at different forecasting times using 35 years of historical weather

**Figure 2:**
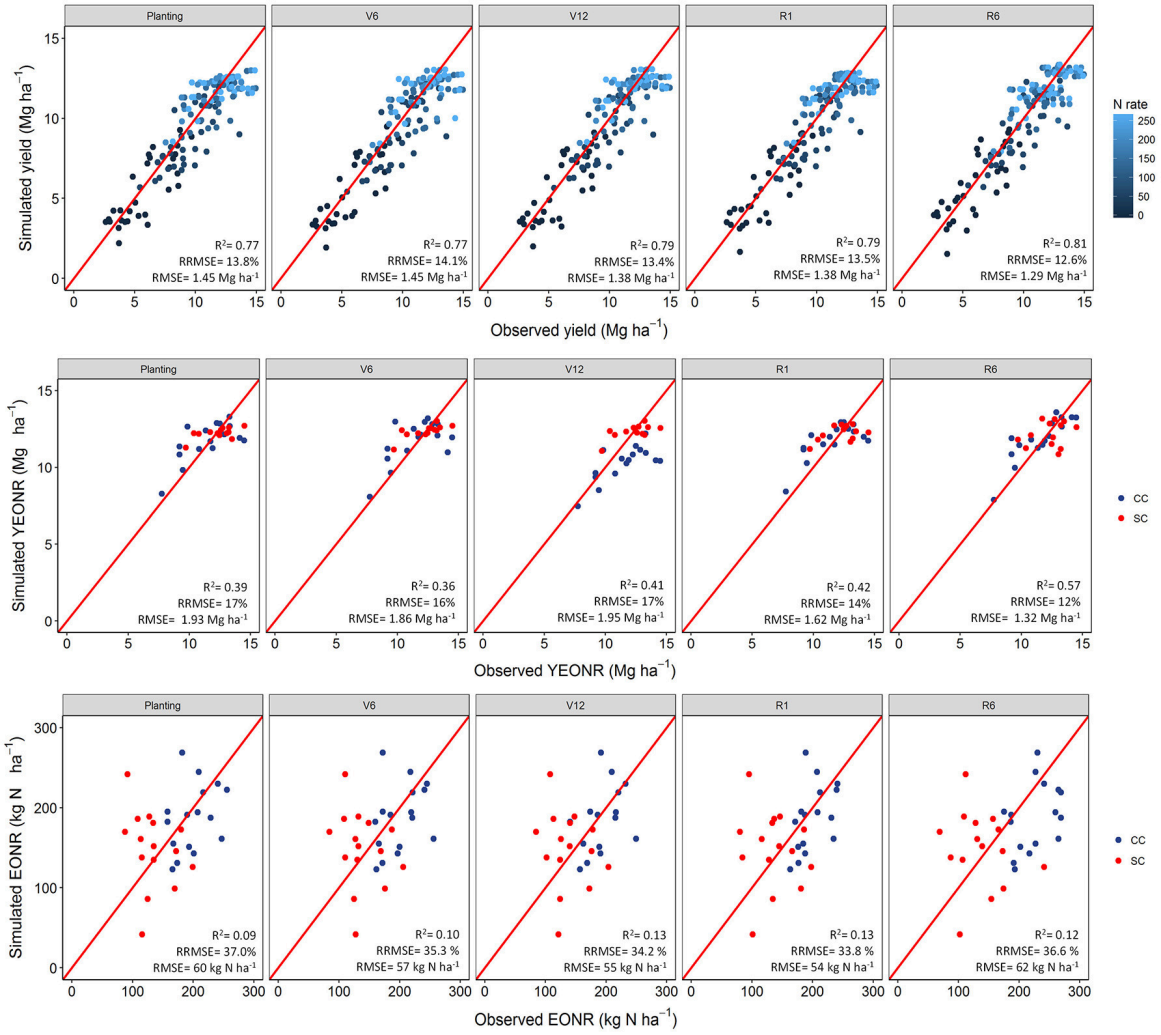
Simulated vs. observed corn yield **(Top panel)**, economic optimum N rate (**Bottom panel**, EONR), and yield at EONR (**Central panel**, YEONR) at different corn stages using 35-yr weather data. In the top panels data presented by N-rate while in the bottom panels data presented by cropping system: CC, continuous corn and CS, soybean-corn. The V6 and V12 are the 6th and 12th leaf stage, respectively; R1 and R6 are silking and physiological maturity stages, respectively (Abendroth et al., 2011).

Yield prediction at different forecast times did not significantly change during the growing season compared with yield prediction at maturity (Figure 2). On average, the RRMSE decreased by 1.2% from planting to maturity. Across five N rates, two crop rotations, and 16 years APSIM explained 77% of the observed variability of corn yield (Figure 2) when predictions were made early in the season (planting and V6 stage), about 79% of the variability during mid-season (V12 and R1 stages), and 81% of the variability at the end of the season (R6 stage). The simulated mean yield at the four forecasting times (with unknown weather after the corresponding growth stage) was similar to the final yield prediction at R6 simulated with known weather (Figure 2).

At early stages (planting to V12), APSIM was able to explain ~40% of the variability in observed YEONR and 57% of the variability at maturity (Figure 2). The APSIM model was better at explaining the observed variability in the YEONR during warm weather years than cold weather years across the different forecasting times (*R*^2^ ~ 0.8 vs. 0.5, respectively; Table S3). Absolute differences between observed and predicted YEONR were 1.55 and 0.63 Mg ha^−1^ for cold and warm weather years, respectively. Overall model performance improved (*R*^2^ = 0.6; data not shown) when looking at this relationship while excluding the 2008, 2010, 2012, and 2014 extreme weather condition years in the 16-years of experiments (Figure S2).

**Figure 3:**
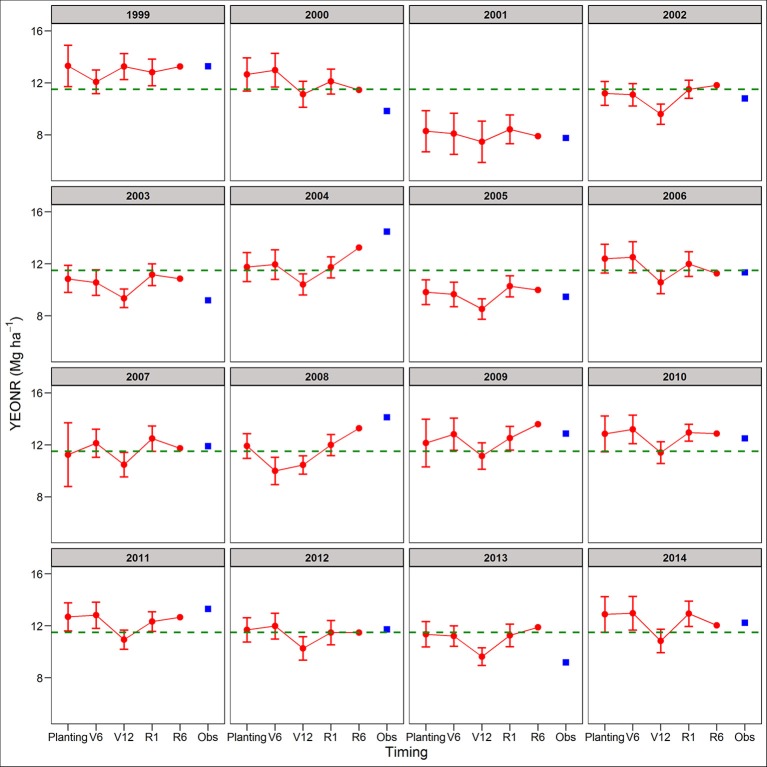
Predicted and observed yield at the economic optimum N rate (YEONR) for continuous corn. The connected red dots indicate APSIM model predictions at planting time, V6 (6th leaf), V12 (12th leaf), and R1 (silking) stages using actual and historical weather data (vertical bars indicate standard deviation, *n* = 35). At maturity (R6), the weather is known so there is no uncertainty in yield prediction. Blue squares represent the observed YEONR. The green dashed line represents the mean YEONR for the study period at this site.

**Figure 4:**
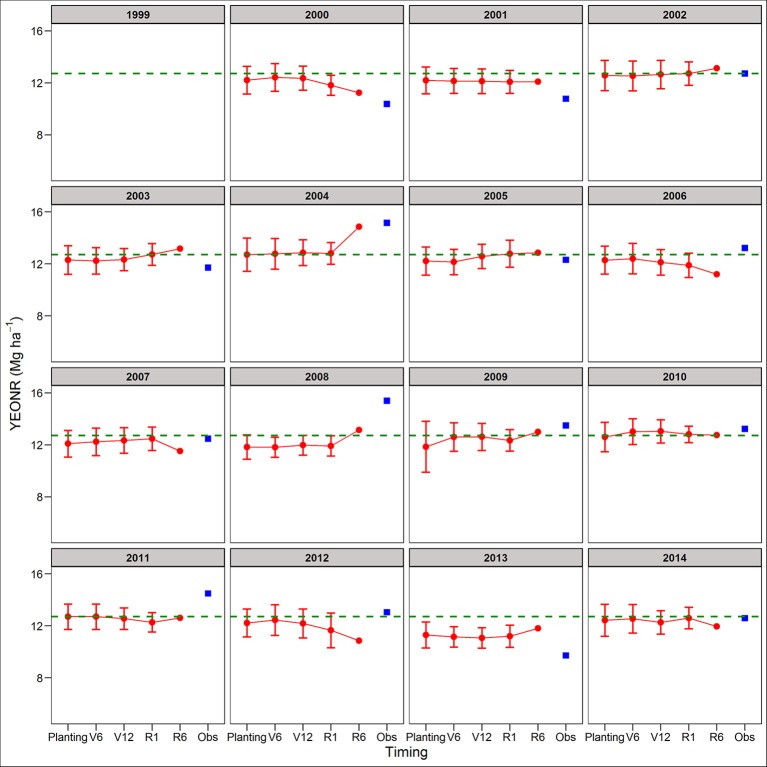
Predicted and observed yield at the economic optimum N rate (YEONR) for soybean-corn. The connected red dots indicate APSIM model predictions at planting time, V6 (6th leaf), V12 (12th leaf), and R1 (silking) stages using actual and historical weather data (vertical bars indicate standard deviation, *n* = 35). At maturity (R6), the weather is known so there is no uncertainty in yield prediction. Blue squares represent the observed YEONR. The green dashed line represents the mean YEONR for the study period at this site.

Between the two cropping systems, yield predictions were slightly better for SC than CC rotations across all four forecasting times (RRMSE of 12.9 vs. 14.2%, respectively; Table S1). The standard deviation of the mean yield, a measure of the uncertainty around yield predictions, did not decrease from planting to R1 (Figures S3, S4). At maturity, there is no uncertainty (standard deviation = 0) because the actual weather was known (Figures S3, S4). In agreement with yield predictions, the uncertainty around the predicted mean YEONR did not decrease from planting to R1 in a significant number of the years (Figures 3, 4). However, the mean deviation (precision) of yield predictions across all years was 27% lower for R1 than at planting (Figures 3, 4).

### EONR prediction at different forecasting times using 35 year of historical weather

**Figure 5:**
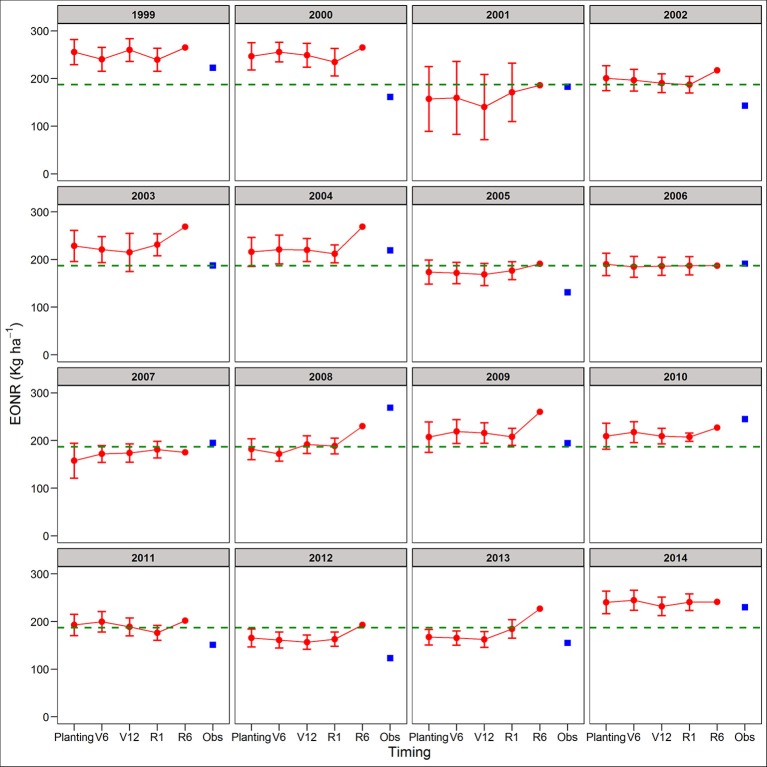
Predicted and observed economic optimum N rate (EONR) for corn-corn. The connected red dots indicate APSIM model predictions at planting time, V6 (6th leaf), V12 (12th leaf), and R1 (silking) stages using actual and historical weather data (vertical bars indicate standard deviation, *n* = 35). At maturity (R6), the weather is known so there is no uncertainty in yield prediction. Blue squares represent the observed EONR. The green dashed line represents the mean EONR for the study period at this site.

**Figure 6:**
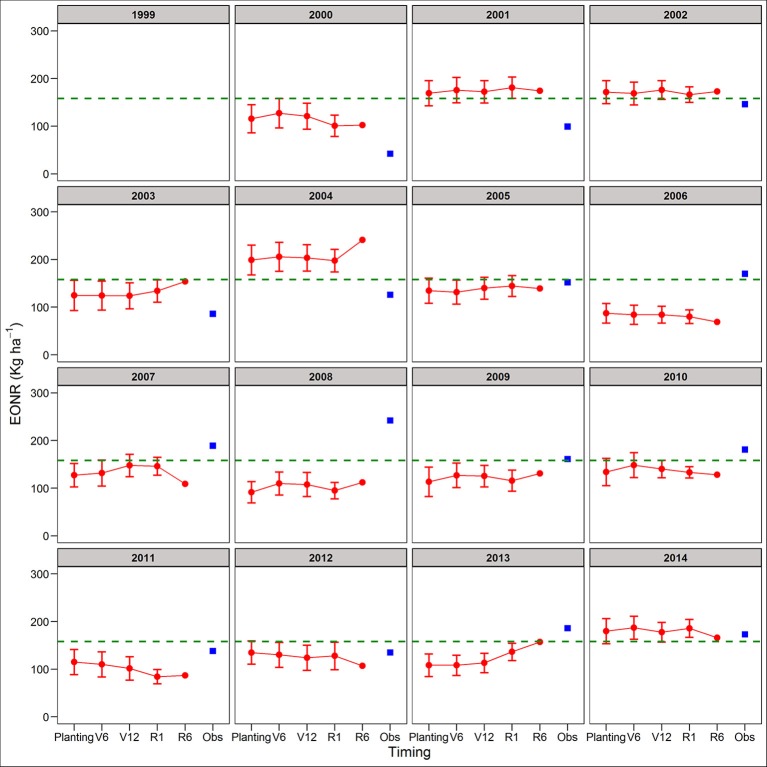
Predicted and observed economic optimum N rate (EONR) for soybean-corn. The connected red dots indicate APSIM model predictions at planting time, V6 (6th leaf), V12 (12th leaf), and R1 (silking) stages using actual and historical weather data (vertical bars indicate standard deviation, *n* = 35). At maturity (R6), the weather is known so there is no uncertainty in yield prediction. Blue squares represent the observed EONR. The green dashed line represents the mean EONR for the study period at this site.

In contrast to yield predictions, the EONR predictions were less accurate (Figure 2), and at different forecasting times showed different patterns across rotations and years (Figures 5, 6). In some cases, the EONR prediction was more accurate at planting or V6 than at maturity (e.g., 2004, Figure 5). In other cases, the EONR prediction was more accurate at maturity than early in the season, but the differences in terms of actual value were small (e.g., Figure 5, 2007, the difference between simulated EONR at planting and maturity was 18 kg N ha^−1^ for CC). Overall, the mean deviation (precision) of EONR predictions across all years was 25% lower for R1 than at planting (Figures 5, 6). The forecasted site-mean EONR and the standard deviation was similar to the observed values, but low for SC and high for CC (Table 1).

**Table 1:** Site-mean and the associated standard deviation economic optimum N rate (EONR, units: kg N ha^−1^) for each crop rotation, forecast time, and the measured site-mean across years.

	**Soybean-Corn**	**Corn-Corn**
Simulated site-mean at planting (*n* = 560)	135 ± 42	199 ± 43
Simulated site-mean at 6th corn leaf (*n* = 560)	138 ± 41	200 ± 42
Simulated site-mean at 12th corn leaf (*n* = 560)	137 ± 39	193 ± 40
Simulated site-mean at corn silking (*n* = 560)	135 ± 40	199 ± 43
Simulated site-mean at corn maturity (*n* = 16)	137 ± 43	225 ± 33
Measured site-mean (*n* = 16)	149 ± 48	188 ± 42

At early forecasting times (planting, V6, and V12), the absolute average differences between observed and predicted EONR for both rotations were lower for warm than for cold weather years (37 vs. 50 kg N ha^−1^, respectively; Table S3). The model better explained observed variability in the EONR in cold/dry than warm/wet seasons across forecasting times and crop rotations (*R*^2^ = 0.32).

In terms of uncertainty, the standard deviation of the mean EONR prediction did not show a consistent pattern of decrease during the growing season (Figures 5, 6). Overall, the EONR predictions were more accurate in CC (RRMSE = 25%) than SC (RRMSE = 45%) across forecast times (Figure 2).

The APSIM model predicted EONR with an average error of ±38 kg N ha^−1^ in 62% of the study cases (*n* = 31). Prediction error was below the threshold value of ±30 kg N ha^−1^ in about 50% of the cases.

### Assessing the impact of different weather scenarios

**Figure 7:**
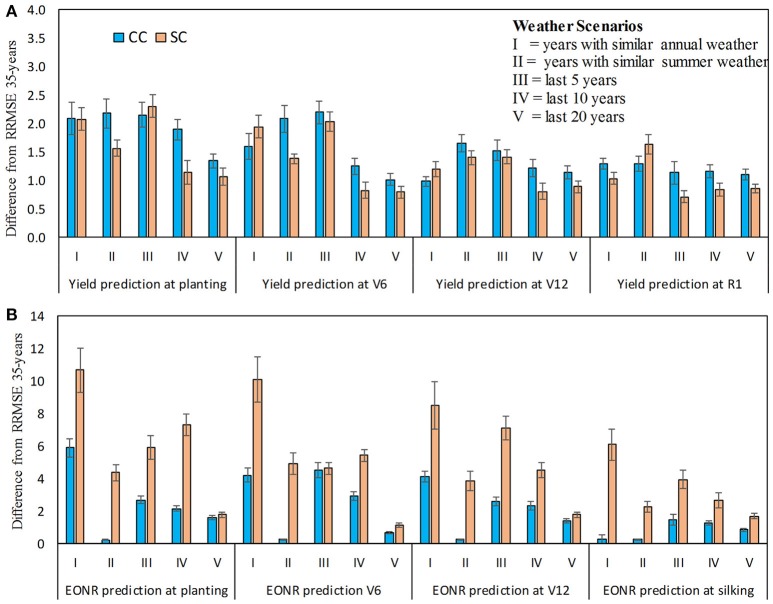
Differences in relative root mean square error (RRMSE) between weather scenarios (I-V) and standard approach (use of 35-years of weather data) for predicted corn yield **(A)** and economic optimum N rate **(B)** at four forecasted times: planting, V6 (6th leaf), V12 (12th leaf), and R1 (silking) stage. The positive values indicate that using the 35-years of weather history is better than use of other weather scenarios (I–V). Continuous corn (CC) and soybean-corn (SC). Vertical bars represents the standard deviation.

Using 35 years of weather data as input to the model resulted in marginally lower RRMSE (on average 1.4%) values and therefore better yield predictions across forecast times compared to the use of 5, 10, or 20 years of weather data or weather data that included only years with similar weather patterns (Figure 7A). Similar results were found for the EONR prediction; that is, use of 35-year weather data performed better than all other scenarios (Figure 7B). Across all forecasting times, RRMSE was 3.5% lower when using 35-years of weather data. Among the scenarios compared against the use of 35-years, the use of 20 years of weather data had the lowest RRMSE for yield and EONR at all forecasting times (Figure 7A). The exception was the yield predictions at V12 and silking stage where the RRMSE was the lowest for scenarios with 5, 10, or 20 years of weather data (Figure 7A).

Independent of the weather scenarios, RRMSE values for EONR predictions were four times higher compared to yield predictions. The selection of a specific weather scenario tended to have a greater impact on predictions early in the season than toward the end-of-season (Figure 7). For example, at flowering the RRMSE was on average across weather scenarios 0.8 and 2.3% lower than RRMSE at planting for yield and EORN, respectively.

### Comparison between APSIM and the 16 year site-mean EONR and YEONR

We use the site-mean EONR and YEONR (see green horizontal line in Figures 3–6) to benchmark the direction and magnitude of error in annual APSIM predictions. Across 16-years in CC and 15-years in SC (*n* = 31 cases), the observed EONR values were in 11, 14, and 6 cases above, below and at site- mean EONR values respectively. APSIM predictions of EONR at planting time were in 11, 16, and 4 cases, above, below and at the site-mean EONR values respectively (Figures 5, 6). In 19 of 31 (62%) cases, APSIM correctly predicted the direction of annual EONR being above, below, or at average. For those cases, the average error was 38 kg N ha^−1^ which represents 20 and 25% error base on the average N rate for CC (observed mean = 184 kg N ha^−1^) and SC (149 kg N ha^−1^), respectively.

The observed YEONR values were in 14, 11, and 6 cases above, below and at site-mean YEONR values respectively, across rotations and years. APSIM predictions of YEONR at planting time were in 8, 14, and 9 cases, above, below and at the site-mean YEONR values respectively (Figures 3, 4). In 21 of 31 (67%) cases, APSIM correctly predicted at planting time the direction of annual YEONR being above, below, or at average.

**Figure 8:**
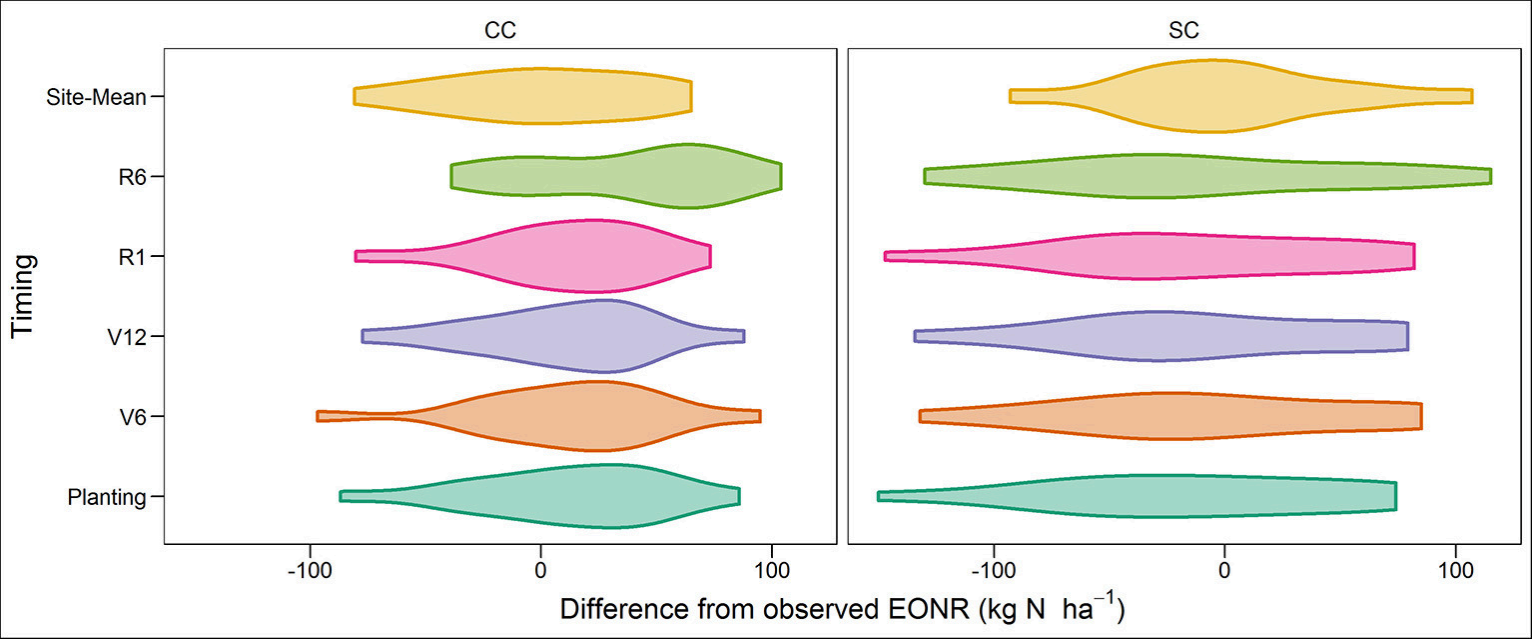
Distribution of differences between simulated economic optimum N rate (EONR) at planting time, V6 (6th leaf), V12 (12th leaf), R1 (silking), and R6 (maturity) growth stages, and the site-mean EONR minus the yearly observed EONR for continuous corn (CC) and soybean corn (SC) rotation.

In low or high N need years the simulated EONR deviated more from observations than in average N need years (Figures 5, 6; Table 2). In 2 out of 16 years (extreme wet years; 2008 and 2010) the APSIM model greatly underestimated the EONR when > 30 kg N ha^−1^. In low fertilization need years, APSIM over predicted the N rate on average by 36 and 69 kg N ha^−1^ in CC and SC rotation, respectively (Table 2). Overall the distribution of the differences between APSIM predicted EONR and observed EONR was skewed to the right (~ + 30 kg N ha^−1^) for CC and skewed to the left for SC, underestimating the observed EONR (Figure 8).

**Table 2:** Comparison of economic optimum nitrogen rate (EONR) estimated by APSIM to the site mean EONR and observed EONR for continuous corn and soybean-corn over three categorical EONR ranges.

**Timing**	Categorical EONR ranges^*^	**APSIM-Observed annual EONR**	
		**Corn-Corn**	**Soybean-Corn**
		**--------------------kg N ha^−1^------------------**	
Planting	High N need year (30 kg N ha^−1^ above the measured site-mean EONR)	−16	−84
V6		−18	−74
V12		−15	−72
R1		−19	−71
R6		9	−73
Planting	Average year (± 30 kg N ha^−1^ from the measured site-mean EONR)	13	−8
V6		17	−7
V12		11	−8
R1		17	−12
R6		38	−11
Planting	Low N need year (30 kg N ha^−1^ below the measured site-mean EONR)	40	61
V6		38	67
V12		33	63
R1		37	63
R6		65	68

* *Observed categorical ranges for the EONR based on site-mean of 188 (corn-corn) and 149 kg N ha^−1^ (soybean-corn)*.

## Discussion

We demonstrated an alternative way to utilize the power of mechanistic cropping systems models to assist N-rate decisions in real time as opposed to current ex-post use of models that are of little interest to farmers (Quiring and Legates, 2008; Thompson et al., 2015). This is the first study that concurrently forecast corn yields and their N requirements in the USA Corn Belt.

### Yield forecasts

This study showed that end-of season corn yields and YEONR can be predicted within an acceptable error (RRMSE < 17%) as early as planting time in most of the study years (Figures 2–4 and Figures S3, S4). The 16-years of data used to test our yield forecasts accounted for different weather years (wet, dry, warm, cold, Figure S1a) and management practices, which further increased our confidence that our process-based forecasting approach is robust (He et al., 2017). This means that our science-based yield forecasting approach has the potential to inform corn producers in the USA Midwest that are using the yield-goal approach to predict N-rates by “guessing” end-of-season yields (Arbuckle and Lasley, 2013; Raun et al., 2017; Morris et al., 2018). Further testing of our approach across multiple environments is needed before application. This would minimize expected errors that are often encountered when using a conventional yield approach based on yield averages across years (Raun et al., 2017).

In most cases, the simulated yield at maturity was within the standard deviation of yield prediction at planting (Figures S3, S4). The uncertainty in yield predictions did not substantially decrease from planting to silking even though that the weather uncertainty decreased (Figure S3). We believe that if we had run the forecast at 150°C-days (or 15 calendar days) after silking we would have seen a decrease in the uncertainty as two of the major determinants of corn yield (kernel number and potential kernel weight) is set (Andrade et al., 1999; Borrás and Vitantonio-Mazzini, 2018). Previous studies have shown a decrease in the uncertainty around corn yield predictions at or about 150°C-days after silking (Thornton et al., 1997; Hansen et al., 2004; Togliatti et al., 2017). By R1 stage, about 50% of the weather is still unknown and the weather during grain fill period has substantial effects on yield. This weather uncertainty introduces yield variability in model predictions as different weather variables affects various plant and soil processes (and thus the final product that is yield) in different ways that are also phenologically time dependent (Semenov et al., 1993). For a thorough sensitivity analysis of weather effects on APSIM simulated grain yield for central Iowa, USA we refer to Togliatti et al. (2017).

In two of the years, yield predictions at maturity deviated substantially from the simulated yields at planting (2004 and 2008 in both rotations; Figures 3, 4). In these years, yield predictions changed during the season as a response to extremely low temperature conditions during grain fill compared to the 35-year average temperature (Figures S2A, S5). The model responded to this weather event by increasing the length of grain fill resulting in higher yield predictions at maturity than at early stages. However, in our study the response of the model was not enough to match the observed yields; perhaps use of a newer maize version (Soufizadeh et al., 2018) of the APSIM model may capture these dynamics better. Nevertheless, this example shows that use of crop model offers both predictability and the reasons behind yield predictions (Banger et al., 2017).

### EONR forecast

The APSIM model predictions at planting time were directionally correct in 62% of the study cases. This means that our N forecasting approach is promising and has future potential to directly and/or indirectly aid N rate decisions by providing a year-to-year opportunity to adjust N rate recommendations early in the season (Figures 5, 6). In terms of prediction accuracy, our modeling approach forecasted annual EONR values at all forecast times with an error range of ±38 kg N ha^−1^ in about 62–69% of the simulated cases (Figures 5, 6, 8). These results emphasize the difficulty in predicting annual EONR in corn production. Given a potential threshold error range of ±30 kg N ha^−1^, this means that further improvements are needed in modeling algorithms as well as development of more precise soil and crop management inputs to the model.

To our knowledge, there is no other tool that can provide both yield and EONR forecasts as early as at planting time. The soil nitrate test and remote sensing approaches are in-season tools. Pre-season tools such as the yield-goal approach rely on yield guesses (Van Es et al., 2006; Sela et al., 2017); while the MRTN tool incorporates N response variation and accounts for price fluctuations, but does not directly adjust for year-to-year variability (Sawyer et al., 2006).

As illustrated in Figure 1, there are many dynamic and interactive factors involved in EONR prediction (Scharf, 2001; Mamo et al., 2003; Scharf et al., 2006; Dhital and Raun, 2016). Every year, management, environment, and genotype interactions determine the shape of the yield response to N fertilizer relationship that is used to estimate EONR. Our modeling approach accounted for the majority of the factors by simulating residue decomposition, soil organic matter dynamics, changes in soil temperature, moisture, and nitrate levels during fallow periods and accounted for management such as planting date, row spacing and date of N application. All these factors affect soil N supply and crop N demand. Low EONR predictions from the model were usually associated with high estimates of soil residual nitrate levels at planting and delays in planting. But, there are many interactions that simultaneously occur among soil-plant processes within the system that makes it difficult to simplify and generalize the reasons of yield and EONR variability (see Figure S5 and Table S2). For example, in a previous work using APSIM model, we found that spring precipitation was related to N losses and EONR (the higher the spring precipitation the higher the EONR; Puntel et al., 2016). In contrast, a later study showed that the amount of precipitation had little influence on simulated grain yield in central Iowa because of the effect of groundwater tables (Togliatti et al., 2017).

The inter-annual prediction accuracy of the EONR forecast was lower than that of the yield forecast (Figures 3–fig66, Figures S3, S4). One reason is that EONR values are not a direct output of the model but a result from a regression analysis of model outputs (yields) which incorporates further uncertainty (Puntel et al., 2016). Across years, the simulated site-mean EONR value by the model was similar to the observed site-mean (Table 1). We believe that by running the model sequentially, annual over-and under-predictions are canceled out and the model is able to predict the site-mean values more accurately.

### Future improvements toward more accurate N rate forecasts

This study identified three areas for future research: (1) simulation set up, continuously vs. annual reset; (2) YEONR and EONR predictions in extreme years, and (3) weather data to drive simulations.

Our simulation protocol is characterized by a high degree of difficulty as we ran the model sequentially from 1999 to 2014 to avoid annual re-initialization of soil input parameters and to capture the carry-over effects on N dynamics (Constantin et al., 2011; Basso and Ritchie, 2015). That approach was followed because of the lack in data to update the model every year and because there was evidence from other studies that models can simulate yields and organic matter trends at different N rates in the long term (Ma et al., 2007; Puntel et al., 2016). The drawback of this approach is that simulation errors can accumulate over time and affect the next year's simulation, especially if the model fails to predict crop yield and N dynamics in one of the years (Salo et al., 2016).

We believe that yield and EONR forecasts at planting time can be further improved if additional information on soil nitrate, water, and surface residue was available to eliminate model uncertainties in simulating carry-over effects during fallow periods (Hansen et al., 2006; Carberry et al., 2009; Ines et al., 2013; Yin et al., 2017). Previous modeling work in Iowa has shown that the available soil water and N status at planting time largely affected predicted mean yields and the range of yield level probabilities (Archontoulis et al., 2016). Therefore, when the model is primarily used for accurate year-by-year yield and EONR forecasting purposes, an annual reset approach may be more appropriate than sequential (Ines et al., 2013; Iqbal et al., 2017). This information can be derived from emerging technologies such as remote sensing, and other common tools such as soil N testing (Basso et al., 2001; Jin et al., 2017; Reimer et al., 2017). Testing and possible improvements in the APSIM surface organic matter model for corn and soybean residues in future studies may improve the simulation of the carry over effects and predictions of EONR, especially in the SC rotation.

There is a need for further research to improve the annual EONR predictability in extreme weather years with precipitation above or below historical average precipitations. Differences between predicted and observed EONR tended to be higher for cold weather years (temperature below the historical average), and their agreement was largely affected by the inaccurate prediction on extremely wet (i.e., 2008) and dry (i.e., 2000) weather years (Figure S2). In this study, we noticed that EONR forecast was more accurate in years that had EONR values near the long-term site average and the prediction accuracy decreased in years with extreme high or low EONR measured values (±20% from long-term site average, Table 2).

For the farmer, both extreme high or low EONR predictions result in economic loss, with the greatest being yield loss when under-fertilizing in high N rate requirement years. Furthermore, when high N is predicted in low N need years the risk of N loss to the environment is greatest (Raun et al., 2017). In particular, the model failed to forecast accurate EONR values in extreme high precipitation years such as 2008 and 2010. We believe the main reason is the incorrect simulation of the carry-over effects from previous year (i.e., soil inorganic nitrogen, soil moisture, root and carbon and nitrogen inputs from previous crops) or possible underestimation of N losses from the system (He et al., 2017; Yin et al., 2017). Prediction of EONR is very complex and less accurate than yield (Figure 2). Perhaps an alternative way of using the model as a forecast tool may be to predict the key-components of the EONR such as yields and soil supply and plant N uptake dynamics, in which the model performs well (Archontoulis and Licht, 2017). In that way, we avoid accumulation of errors that leads to a low prediction accuracy of the EONR.

For weather uncertainty affecting yield and derived EONR forecasts (Hansen et al., 2006), our results indicated that use of the entire historical record (35 years) in the simulation process is better than selecting years based on similar weather events or using only a smaller set number of previous years (Figure 7). This was more evident in EONR predictions and less in yield predictions. Although the reasons are not sufficiently clear, based on other studies, we believe that simulated crop growth depends more on the distribution of weather within the season than the season average which was used to categorize the years used for the weather scenarios (Hansen and Indeje, 2004; Figure S1b).

We also found that the use of at least 10 and 20 years of weather data is associated with small error (2.5% less accuracy than the 35-year; Figure 7) which is an important finding given that not all sites have accurate weather records for 35 years (Hansen et al., 2004; Grassini et al., 2015). Our findings agree with Van Wart et al. (2013), who showed that 6–10 years of weather data is needed for sites with similar rain patterns as the one used in this study. However, we also recognize there are other ways of selecting years to be used in forecasting studies and this is a topic for further investigation (Hansen et al., 2004).

## Conclusions

This study provided evidence that use of a calibrated cropping systems model can aid yield and N rate forecasts. At planting time, model predictions were directionally correct in predicting whether the optimum N-rate for corn would be above, below or at site-mean value. The associated prediction error was within an error range of ±30 kg N ha^−1^ in ~60% of the years. Predictability of corn yields was more accurate than optimum N-rates at planting time. In most years, in-season yield and optimum N-rate forecasts were not better than the predictions made at the beginning of the season. Use of 35-year historical weather data was found to be more accurate than using analogous weather years based on similarity of current weather conditions. Process-based modeling forecast of corn yields and optimum N-rates is already promising and has potential for realizing further improvements in achieving even more accurate early-season predictions.

## Author contributions

LP, JS, and SA: conceived the research idea and designed the modeling study; LP and SA: performed the model analysis and drafted the manuscript; All coauthors (JS, MC, PT, KM, EH, AV) reviewed the manuscript and contributed to the final version.

### Conflict of interest statement

The authors declare that the research was conducted in the absence of any commercial or financial relationships that could be construed as a potential conflict of interest.
