# Supplementary material for: A Systems Modeling Approach to Forecast Corn Economic Optimum Nitrogen Rate
Source: Front Plant Sci. 2018 Apr 13;9:436. doi: 10.3389/fpls.2018.00436 (PMC5909184; doi:10.3389/fpls.2018.00436)
Supplement: Supplementary file 1 [file DataSheet1.pdf]

Supplementary material

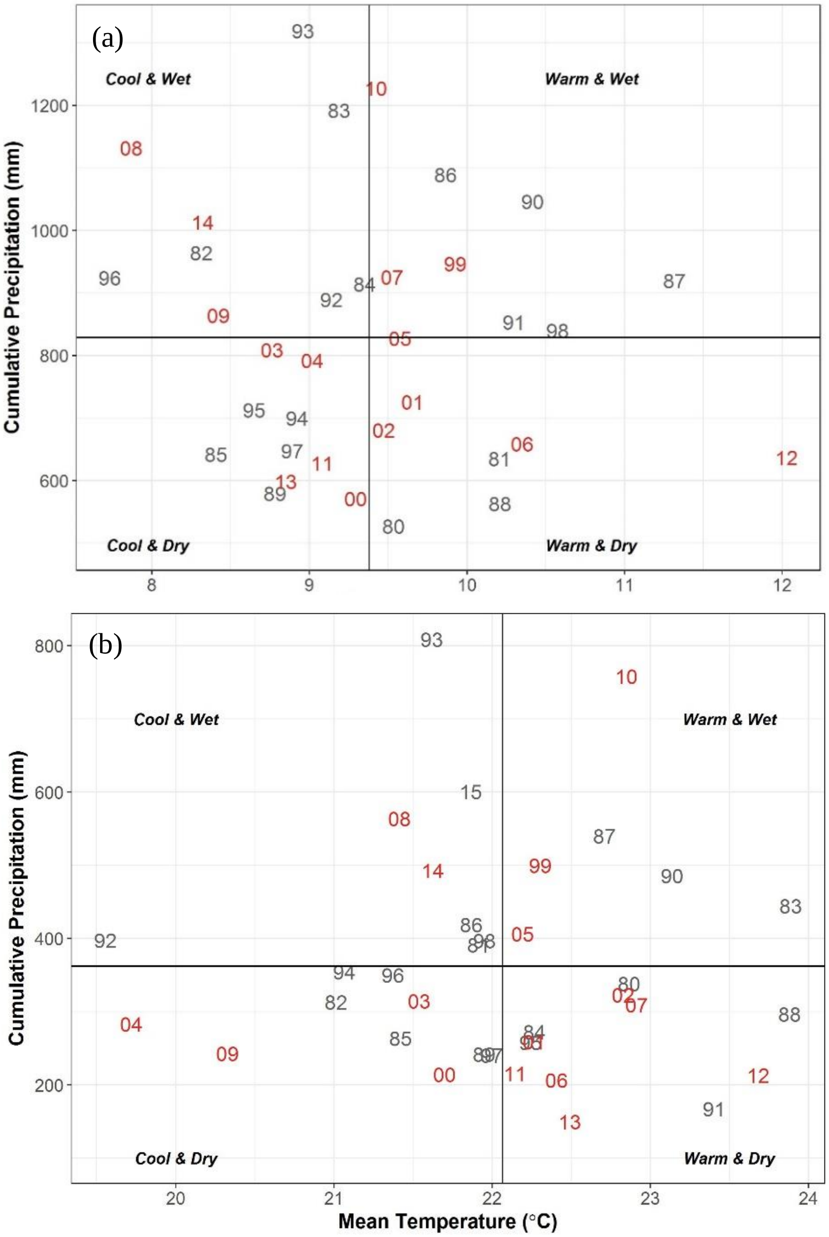

Figure S1. Cumulative annual (a) and summer (June to August, b) precipitation and mean temperature in Ames, Iowa, USA. The long-term average cumulative precipitation and temperature across years (1980-2014) are shown with the vertical and horizontal lines, respectively. These average values were used to classify years into warm, cool, dry, and wet. Years shown in red represent the years used in this study (1999–2014).

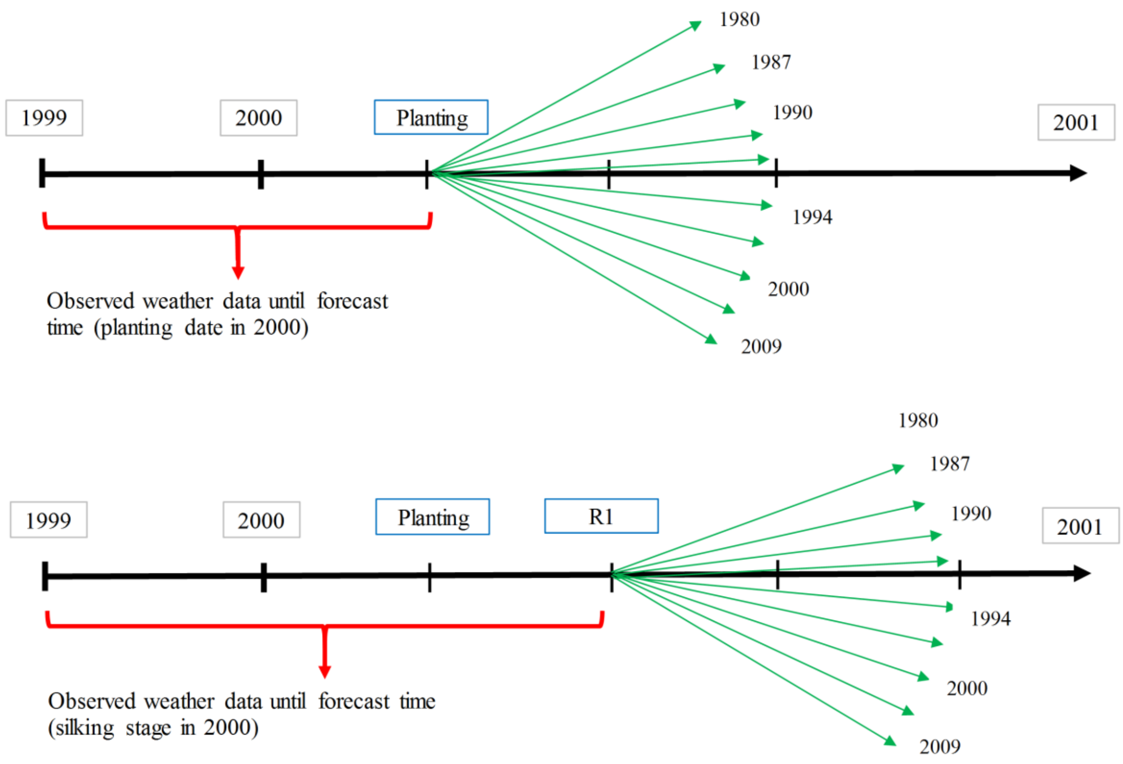

Figure S2. Example of the methodology used to assemble synthetic weather files to run the APSIM model at different forecasting times. Synthetic weather files have known weather until the time of the forecast (e.g. planting, see top panel or flowering time see bottom panel) and multiple historical weather data (35 years) until the end of a particular growing season.

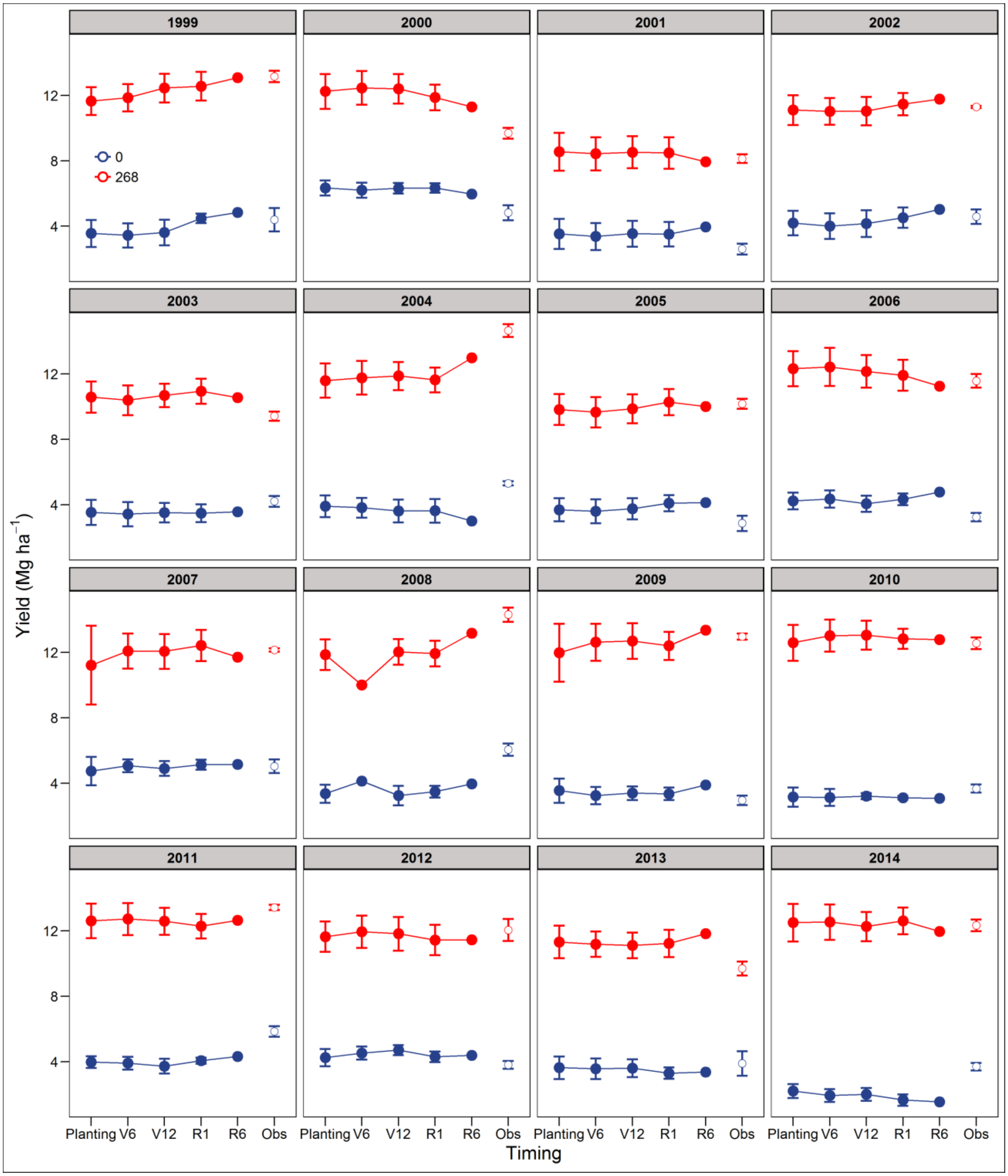

Figure S3. Simulated corn yields at various corn growth stages for continuous corn at 0 and 268 kg ha^{-1} applied N rate. Simulations performed for planting time, V6 (6th leaf), V12 (12th leaf), and R1 (silking) stages (Abendroth et al., 2011) using actual and historical weather data (n=35; vertical bars indicate standard deviation). At R6 (maturity) stage the weather was known (n=1). The observed corn yields at harvest (Obs) are also shown (n=4 replications).

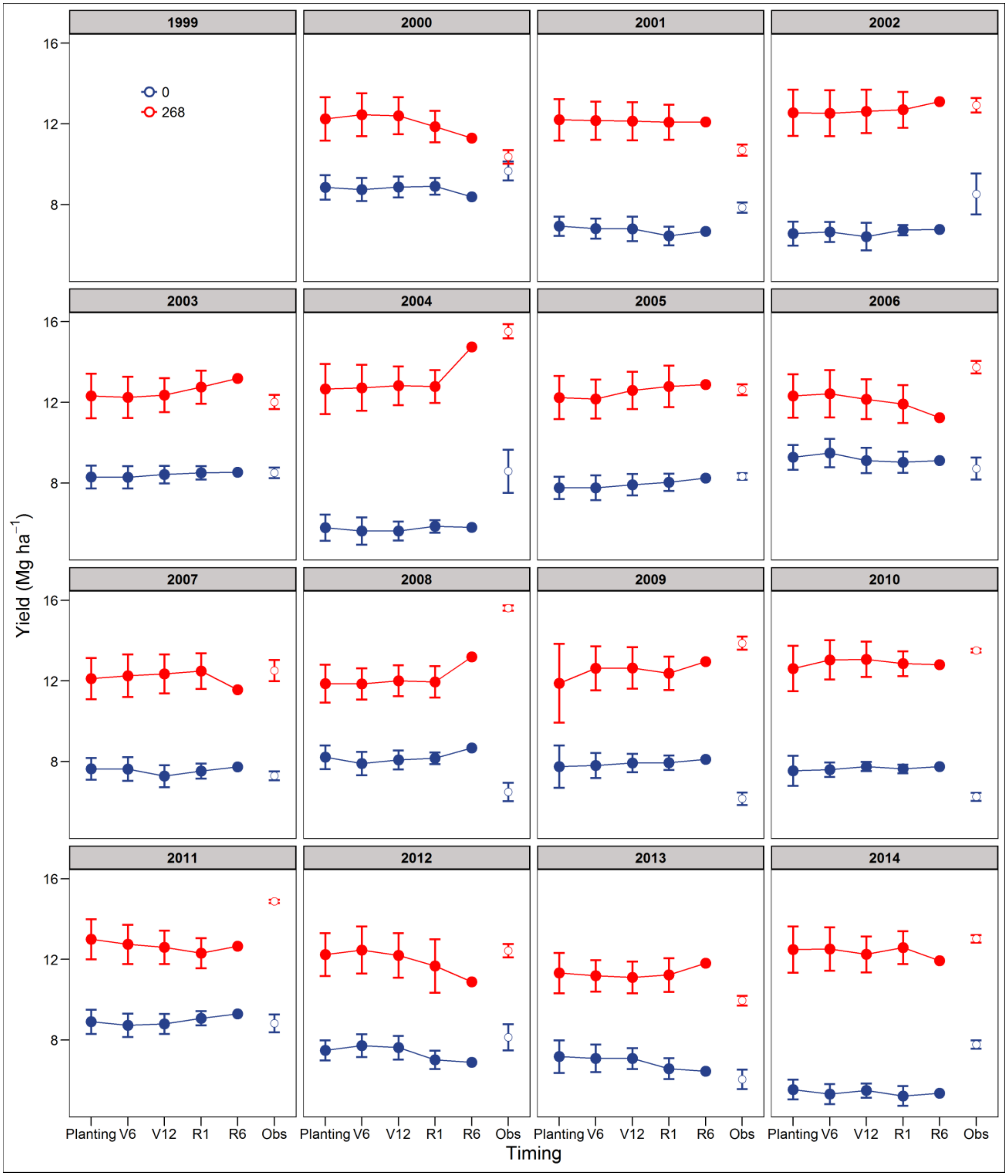

Figure S4. Simulated corn yields at various corn growth stages for soybean-corn at 0 and 268 kg ha^{-1} applied N rate. Simulations performed for planting time, V6 (6th leaf), V12 (12th leaf), and R1 (silking) stages (Abendroth et al., 2011) using actual and historical weather data (n=35; vertical bars indicate standard error). At R6 (maturity) stage the weather was known (n=1). The observed corn yields at harvest (Obs) are also shown (n=4 replications).

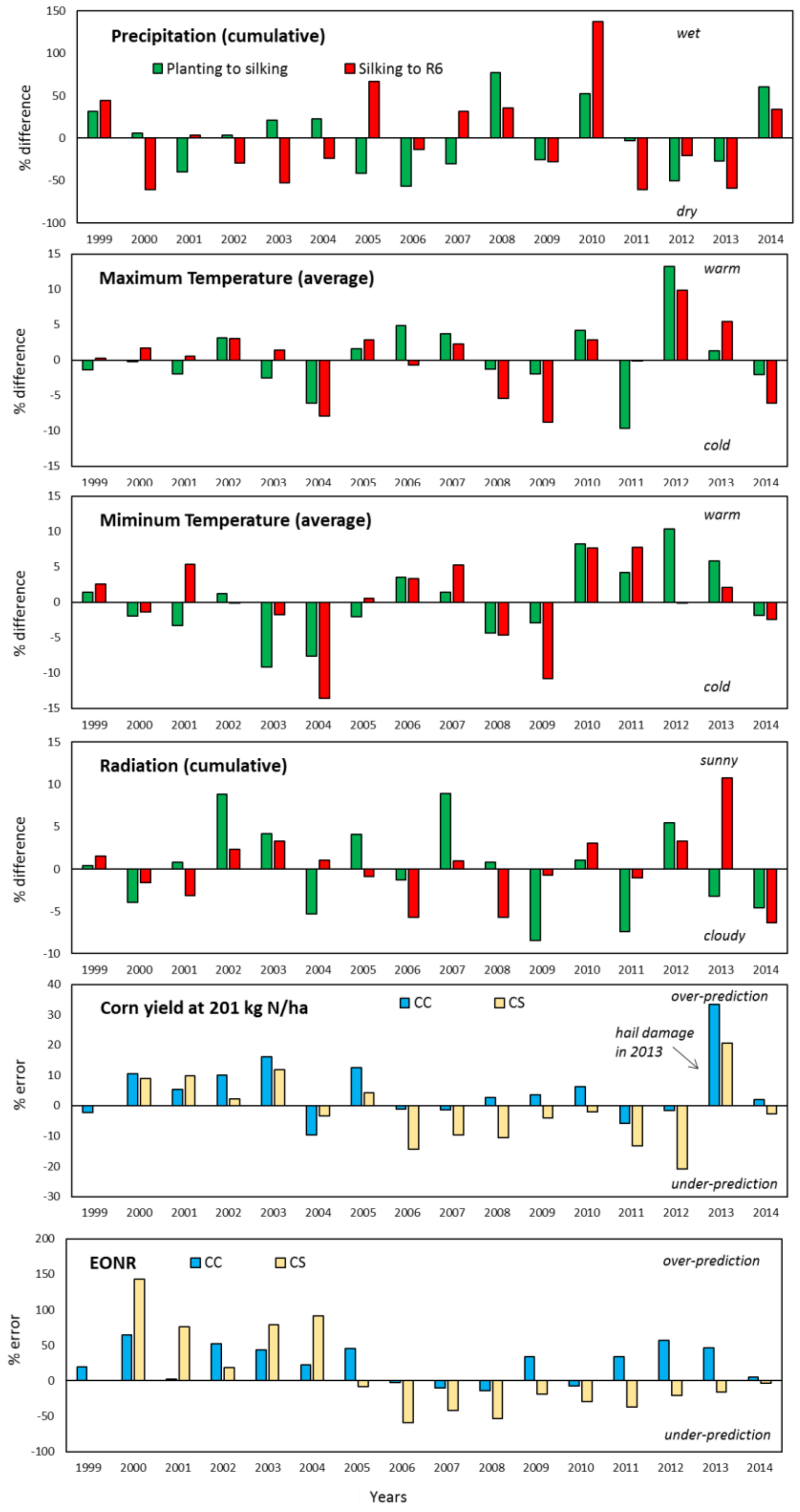

Figure S5. Precipitation, maximum and minimum temperature, and radiation differences from the historical average. The lowest two panels show percent error between simulated and observed corn yield at 201 kg N ha^{-1} and the economic optimum nitrogen rate (EONR) for continuous corn (CC) and soybean-corn rotation (SC).

Table S1. Root mean square error (RMSE) and relative RMSE (RRMSE) for simulated corn yields at continuous corn (CC) and soybean-corn (SC) at different forecasting times.

Rotation	Timing*	RMSE	RRMSE
		---------kg ha^{-1}-----------	
	Planting	1358	14.7
	V6	1443	15.6
CC	V12	1301	14.1
	R1	1279	13.9
	R6	1152	12.4
	Planting	1475	13.0
	V6	1463	12.9
SC	V12	1458	12.8
	R1	1483	13.0
	R6	1430	12.5
Combined	Planting	1416	13.8
	V6	1453	14.2
	V12	1380	13.4
	R1	1382	13.5
	R6	1294	12.6

*Growth stages: planting time, V6 (6th leaf), V12 (12th leaf), R1 (silking), and R6 (maturity); Abendroth et al., 2011.

Table S2. Regression coefficients (R^2) between grain yield or economic optimum nitrogen rate (EONR) error (absolute difference between simulated and observed) and weather deviations of the data presented in figure S3. The year 2013 was excluded from this analysis due to hail damage.

Weather variable	CC		CS	
	Yield	EONR	Yield	EONR
Precipitation (pre-flowering)	0.0003	0.088	0.024	0.0041
Precipitation (post-flowering)	0.0021	0.337* (*p*=0.023)	0.0022	0.1886
Max. temperature (pre-flowering)	0.0025	0.0058	0.114	0.0859
Max. temperature (post-flowering)	0.082	0.128	0.0046	0.0008
Min. temperature (pre-flowering)	0.007	0.0004	0.388* (*p*=0.017)	0.296* (*p*=0.044)
Min. temperature (post-flowering)	0.009	0.05	0.0062	0.096
Radiation (pre-flowering)	0.137	0.0001	0.003	0.0167
Radiation (post flowering)	0.0016	0.198	0.0123	0.0042

* Significant correlation at $p < 0.05$.

Tables S3. Correlation (R^2) and absolute difference between observed and predicted economic optimum nitrogen rate (EONR) and yield at EONR (YEONR) for four categories of weather years.

Weather years	EONR-R^2	EONR-Absolute difference	YEONR-R^2	YEONR-Absolute difference
	-	-----kg N ha^{-1}----	-	----Mg ha^{-1}----
Cold and wet	0.02	49	0.47	1.40
Cold and dry	0.30	51	0.50	1.70
Warm and wet	0.34	34	0.87	0.48
Ward and dry	0.02	40	0.71	0.78
